# Opium in Metro-Peritonitis

**Published:** 1870-12

**Authors:** Theodore Griffin

**Affiliations:** 529 State Street, Chicago


ARTICLE XL.

### OPIUM IN METRO-PERITONITIS.

By THEODORE GRIFFIN, M.D., 529 State Street, Chicago.

Appreciating the fact that it is only by recording and giving to our brethren the results of carefully-observed experience in the treatment of disease, that true and reliable progress is made in therapeutics, I offer for consideration my record of the following case of metro-peritonitis, treated with opium.

I present it briefly and without comment, as a link added to the chain of evidence in favor of the use of opium in this fearful disease. I was led to the employment of large doses by the plain indications to be fulfilled, which, I know the physiological effect of opium would meet, bearing in mind this fact, which I think should become an axiom: *if indications are scrupulously met in treatment, no drug poisoning, with its disagreeable and dangerous perturbations, will occur, howsoever large the dose of the drug may be.* It is a lamentable fact that indications are often *more than met*, as occurred at the close of this case through the ignorance of the nurse. * * *

On the 16th day of June, 1870, I attended Mrs. ——— in labor. She says that after previous labors, numerous clots of blood were always expelled from the uterus; after this labor, none were expelled.

On the 17th, she complained of severe pain over the region of the uterus, especially upon the right side, describing it as sharp and lancinating. She had, on this day, a severe chill; her tongue was covered with a white fur; her bowels were constipated; her pulse 90, and bounding. She had, also, headache and aching of the limbs. These symptoms, however, yielded to a little mild treatment, and on the evening of 19th, she felt, as she expressed it, and as her symptoms indicated,—"quite well."

On the morning of the 20th, at 3 A.M., more lancinating pains began in the uterus and over the abdominal region, accompanied by all the initial symptoms of metro-peritonitis. I immediately directed that she have *one gr. of opium every hour.* The pain continued unabated until 9 o'clock P.M. At this time her appearance indicated great suffering; her nervous system was disturbed and agitated; her tongue coated with a dirty black coat; there was nausea and vomiting, with fetid and dark-colored discharges from the bowels; the slightest touch or motion produced the most intense pain; the abdomen was distended to its size immediately previous to confinement; her pulse was 110. I now gave *three grs. of opium* at one dose, and ordered *one gr. to be given every half-hour.* At 8 o'clock A.M., (21st), her pulse was 94, her tongue was still black, but somewhat improved; no more vomiting nor fecal discharges since my last visit; the pain was yet severe. I now gave *four grs. of opium at one dose*, and ordered *two grs. of opium to be given every half-hour.* At 11½ o'clock A.M., her pulse was 100; the pain was still unabated; no signs of opium poisoning. She has passed no urine since 12 o'clock last night; the mammary secretion was now gone, and pus discharged continuously from the vagina. I ordered *five grs. of opium to be given every hour.*

At 4½ o'clock P.M., (21st), nervous symptoms were better, she had less pain and rested comparatively easy; some subsultus tendinum. I now directed *three grs. of opium to be given every half-hour*, and drew her urine with the catheter. At 10 o'clock P.M. she rested well, there was now no subsultus; she had now some inclination to sleep; her pupils were natural and her pulse 100; continued the opium, three grs. every half-hour. At 11 o'clock P.M. she slept for the first time since her illness began; at this time I left her for the night, directing the nurse to discontinue the opium if she awoke without pain, and with an inclination to sleep. At 2 o'clock A.M., (22d), she awoke, bright, intelligent, and free from pain, and, as she told the nurse, *feeling well.* The nurse, however, continued the opium as before, *three grs.* every half-hour. At 5 o'clock A.M. I was called to her in great haste, and found her narcotized, breathing stertorously with contracted pupils; these alarming symptoms, resulting from the *indications being more than met*, quickly disappeared with appropriate treatment. She soon became conscious, convalescence being established from that moment. There remained some tenderness on pressure over the region of of the uterus, but the distention of the abdomen had entirely disappeared with all the symptoms pertaining to the digestive and nervous systems; her pulse was natural, and her restoration to perfect health was rapidly completed.

The amount of opium given is presented in the following summary:

From 3 o'clock A.M., 20th, to 9 P.M., 18 hours, 18 grs.; from 9 o'clock P.M., 20th, to 8 o'clock A.M., 21st, 11 hours, 23 grs.; from 8 o'clock A.M., 21st, to 11½ A.M., 3½ hours, 18 grs.; from 11½ o'clock A.M., 21st, to 4½ o'clock P.M., 5 hours, 25 grs.; from 4½ o'clock P.M., 21st, to 10 o'clock P.M., 5½ hours, 33 grs.; from 10 o'clock P.M., to 11 o'clock P.M., 1 hour, 6 grs. Total—44 hours, 123 grains.

At 2 o'clock A.M., 22d, 6 grs. The last 6 grains produced the narcotism.

No other drug than opium was administered during the entire course of the disease.

